# Multi-Objective Optimization and Performance Characterization of Asphalt Modified by Nanocomposite Flame-Retardant Based on Response Surface Methodology

**DOI:** 10.3390/ma14164367

**Published:** 2021-08-04

**Authors:** Jiaqi Li, Zhaoyi He, Le Yu, Lian He, Zuzhen Shen

**Affiliations:** 1 School of Civil Engineering, Chongqing Jiaotong University, Chongqing 400074, China; yule_cju@163.com (L.Y.); congmingxiaohe@163.com (L.H.); S54846302@163.com (Z.S.); 2 National and Local Joint Engineering Laboratory of Traffic Civil Engineering Materials, School of Civil Engineering, Chongqing Jiaotong University, Chongqing 400074, China; 3 College of Traffic & Transportation, Chongqing Jiaotong University, Chongqing 400074, China; hzyzwb@cqjtu.edu.cn

**Keywords:** asphalt, flame retardant, response surface methodology, multi-objective optimization, physical properties, flame retardant properties, rheological properties

## Abstract

In order to improve the safety of the tunnel asphalt pavement in the event of a fire, and reduce the deterioration of the low temperature crack resistance of the asphalt by the flame retardant. The research uses aluminum hydroxide (ATH) as a smoke suppressant, diethyl aluminum hypophosphite (ADP) as a flame retardant, and halloysite nanotubes (HNTs) as a synergist to modified styrene-butadiene-styrene block copolymer (SBS) modified asphalt (MA). First, the content of ATH, ADP, and HNTs was used as the response variable. The physical properties (Penetration, Softening point, Ductility) and static flame retardant properties (Limiting oxygen index meter, Ignition point) of the asphalt modified by nanocomposite flame-retardant (HNTs-CFRMA) were the response variables. The response surface methodology was used to design the test, and regression models were established to analyze the influence of flame retardants on the performance of asphalt. Then, comprehensively considering the effects of physical properties and flame retardant properties, the normalized desirability function was used to perform a multi-objective optimization design on the components of the nanocomposite flame retardant modifier to obtain the best flame retardant formula. Finally, the rheological properties of MA, conventional flame-retardant modified asphalt (CFRMA), and HNTs-CFRMA were tested based on Dynamic shear rheometer, Multiple stress creep test, Force ductility tester, and Bending beam rheometer. The performance of flame-retardant and smoke suppression were tested by the Cone calorimeter tests. The result shows that ATH, ADP, and HNTs can enhance the high temperature performance of asphalt, reduce the penetration. The addition of HNTs can increase significantly the softening point and reduce the deteriorating effect of flame retardants on the low temperature performance of asphalt; the addition of ATH and HNTs can improve significantly the flame retardancy of asphalt. Based on the desirability function of power exponent, the formulation of the nanocomposite flame retardant with better physical properties and flame retardant properties is ATH:ADP:HNTs = 3:5:1, and the total content is 9 wt%. Nanocomposite flame retardants can improve obviously the high temperature rheological properties of asphalt. The rutting factor and the cracking factor of HNTs-CFRMA improve markedly, and the irrecoverable creep compliance is reduced, compared with MA and CFRMA. Nanocomposite flame retardant can make up for the deterioration of conventional flame retardants on asphalt’s low temperature performance. At the same time, it has better flame-retardant performance and smoke suppression performance.

## 1. Introduction

In order to meet the higher safety and comfort requirements of long tunnels in mountainous areas and submarine roads in coastal areas, asphalt concrete has gradually replaced cement concrete and become the mainstream of tunnel paving due to its driving comfort, low noise, good skid resistance, and convenient maintenance. However, the asphalt mixture is a combustible material. When a fire occurs in a closed tunnel environment, the asphalt pavement is heated and decomposed, producing a lot of toxic smoke and heat. The decomposition and burning of asphalt pavement will not only accelerate the spread of fire and increase the risk of a fire, but also cause damage to the lining structure and hinder personnel escape and fire rescue [1,2,3,4]. Therefore, the addition of flame retardant has become a common means to improve the fire safety of asphalt pavements. The research on high-performance and environmentally friendly asphalt flame-retardant systems has become a hot spot.

At present, in order to realize the purpose of asphalt flame retardant and improve the fire safety of asphalt pavement, the main method is to add flame retardant to asphalt for physical modification or chemical modification. Common flame retardants are mainly divided into organic flame retardants and inorganic flame retardants. Organic flame retardants can be divided into halogen flame retardants [5], nitrogenous flame retardants [6], and phosphorus flame retardants [7]. Organic flame retardants, mainly halogen flame retardants, have excellent flame retardant properties and can better improve the physical properties of asphalt. However, its use is restricted due to environmental issues such as toxic gases generated when flame retardants decompose. Inorganic flame retardants, mainly metal hydroxide flame retardants [8,9,10], have good flame retardant and smoke suppression properties. However, single flame retardants to improve the flame retardant and smoke suppression performance of asphalt have the disadvantages of large dosage and deterioration of road performance [11]. Researchers began to adopt methods such as multiple compounding of organic and inorganic flame retardants to improve the flame retardant performance of asphalt. Wang et al. [12] compounded the flame retardant max (FRMAX) and aluminum hydroxide magnesium hydroxide phosphate (AMP) to obtain the flame retardant, and passed the oxygen index test (OI) and the combustion test of the Marshall specimen, field combustion test, etc., studied the flame retardant performance of the compounded flame retardant asphalt. Fu et al. [13] analyzed and studied the synergistic flame-retardant properties of decabromodiphenylethane (DBDPE) and antimony trioxide (Sb_2_O_3_), and the compound significantly improved the flame-retardant properties of SBS modified asphalt. Ling et al. [14] selected an intumescent flame retardant system composed of ammonium polyphosphate, pentaerythritol, and melamine. They added the intumescent flame retardant directly to MA to achieve the flame retardant effect. Tan et al. [15] proposed that decabromodiphenylethane, Sb_2_O_3_, zinc borate (ZB), and aluminum hydroxide (ATH) constitute a synergistic flame retardant system. They analyzed the flame retardant effect of the flame retardant, and demonstrated the feasibility of the system. Compared with single flame retardants, the conventional composite flame retardants reduce the cost and improve the flame retardant efficiency. But there are still problems such as large dosage, low flame retardant efficiency of the mixture, and poor low temperature performance. Composite flame retardant materials combined with nanotechnology have already attracted the attention of massive scholars, such as nano montmorillonite (MMT) [16,17,18], expanded vermiculite (EVMT) [19], layered double hydroxides (LDHs) [20,21,22], expandable graphite (EG) [23], and other nanomaterials. But the layered nanomaterials have poor dispersibility and deteriorate the low temperature performance of asphalt, which makes the application of this technology still problematic. In this paper, HNTs is used as a synergist of flame retardancy [24,25]. HNTs is a tubular nanomaterial and is composed of curled layered silicates. It not only has the flame-retardant catalytic effect of nano-montmorillonite, but also has a great dispersion effect in asphalt [26]. The researches [26,27,28] have shown that the mixing of HNTs and conventional flame retardants not only enhance the flame retardant properties of the materials, but also improves the physical properties of the composite materials.

Response Surface Methodology (RSM) is a combination of experimental design and mathematical models to find the most solvable optimization method [29]. RSM has applications in many fields [30,31,32,33,34,35]. Omid et al. [36] applied RSM to obtain the best formula for mixing crushed rubber concrete and older soil to make clean concrete. Because it overcomes the problem that the orthogonal experiment cannot accurately determine the optimal value of each factor, this method can effectively and quickly optimize the formula.

In this work, RSM was used to carry out experimental design and establish response surface models, with the dosage of ATH, ADP, and HNTs as the response variables, and the physical properties of the flame-retardant modified asphalt (Penetration, Softening point, Ductility), flame-retardant performance (Limiting oxygen index, Ignition point) five indicators for the response value. Analyze the influence of various factors on the conventional performance and flame-retardant performance of asphalt through these models, and obtain the asphalt modified by nanocomposite flame-retardant with the best performance. Based on the rheological performance test and cone calorimeter tests (CCTs), the flame-retardant and rheological properties of the asphalt modified by nanocomposite flame-retardant are characterized and analyzed in depth.

## 2. Materials and Methods

### 2.1. Materials

The asphalt was SBS modified asphalt, which was produced by Chongqing Heavy Traffic Renewable Resources Development Co., Ltd., Chongqing, China. The technical indicators of the asphalt meet the specification ASTM [37,38,39,40,41] and AASHTO [42,43,44], as showed in Table 1. The smoke suppressant was ATH, which was produced by Shandong Taixing New Materials Co., Ltd., Qingdao, China. The ATH is 8000 mesh high-purity ultra-fine aluminum hydroxide, the average particle size of which is 1.6–2.6 um, density is 2.4 g/cm^3^ and purity is 99.0%. The flame retardant was ADP, which was produced by Guangdong Wengjiang Chemical Reagent Co., Ltd., Guangzhou, China. The ADP is an analytically pure diethyl aluminum hypophosphite with a molecular weight of 390.27, and its purity is over 98.0%. The flame retardant synergist was HNTs, which was purchased from Guangzhou Yuanxin Nano Technology Co., Ltd., Guangzhou, China. The average particle size of HNTs is 20–100 nm.

### 2.2. Preparation Method and Process of Composite Flame-Retardant Modified Asphalt

A two-step method is used to prepare composite flame-retardant modified asphalt. The first step: pre-shear MA and HNTs in a high-speed shearing machine (HFS-40, KEDE Machinery Co., Ltd., Dongguan, China) at 160 °C and 1500 rpm for 10 min; 5000 rpm high-speed shearing for 40 min; adding stabilizer (0.1%) 500 rpm low-speed shearing for 10 min. The second step: add the conventional flame retardant (CFR) at 1000 rpm and quickly stir for 10 min using a scrub disperser to obtain the HNTs-CFRMA.

### 2.3. Performance Test of Composite Flame-Retardant Modified Asphalt

#### 2.3.1. Physical Properties

Asphalt penetration testing was tested with an SZR-3 asphalt penetration tester (Shanghai Rongjida Instrument Technology Co., Ltd., Shanghai, China), according to ASTM D 5. The high temperature performance testing of asphalt was tested by HR-2806E asphalt softening point tester (Cangzhou Huayi Testing Instrument Co., Ltd., Cangzhou, China), according to ASTM D 113. The low temperature performance testing of asphalt was tested using the LYY-8 ductility tester (Cangzhou Huayi Testing Instrument Co., Ltd., Cangzhou, China), according to ASTM D 36. The test temperature is 5 °C and the tensile rate is 1 cm/min.

#### 2.3.2. Flame Retardant Properties

The limiting oxygen index (LOI) testing of asphalt was tested by JF-3 LOI analyzer (Sanzhong Analysis Instrument Co., Ltd., Chongqing, China), according to ASTM D 2863-19 [40]. The ignition point testing of asphalt was tested using the SYD-3536 Cleveland open cup (Shanghai Precision Instrument Co., Ltd., Shanghai, China), according to AASHTO T 48.

#### 2.3.3. Performance Characterization Analysis

The rheological properties of asphalt were tested with the Bohlin Gemini 2ADS dynamic shear rheometer (Malvern Panalytical, Malvern City, UK). According to AASHTO T 315-2012 [43], adopting the temperature scanning mode for dynamic shear rheology test (DSR-T), the parallel plate size was 25mm and the parallel plate spacing was 1mm. The test adopted the strain control mode, and the strain control was 12%. The frequency was 10rad/s, the test temperature range was 46~82 °C, and the temperature step was 6 °C. The Multiple stress creep test (MSCR) was performed using the multiple stress creep mode. The diameter of the parallel plates used in the test was 25 mm, the distance between the parallel plates was 1 mm, and the stress level was 0.1 kPa and 3.2 kPa. The effects of light and heavy traffic on the creep recovery of asphalt binder were evaluated respectively. The test water bath temperature was 64 °C. Load loading time was 1s, unloading time was 9 s, repeated 10 cycles.

Force ductility tester (FDT) and Bending beam rheometer (BBR) were used to characterize the low temperature performance of asphalt According to ASTM D 36, adopting LYY-8 ductility tester, the test used an ordinary eight-character test model. The test temperature was 5 °C. Before the test, the sample was placed in a water bath at 5 °C for 1.5 h. During the test, the tensile rate was 1cm/min, and the test was stopped until the specimen broke. The TE-BBR bending beam rheometer (CANNON Instrument Co., State College, PA, USA) was used for the test. According to ASTM D 6648-08 [41] and AASHTO T 313-08 [44], the sample size of the test piece was: length 127 mm ± 2.0 mm, width 12.70 mm ± 0.05 mm, height 6.35 mm ± 0.05 mm. The load during the test was 980 ± 50 mN. Took the creep stiffness (S) and creep rate (m) at −24 °C, −18 °C, and −12 °C when the trabecular was bent for 60 s.

The heat release characteristics and smoke release characteristics of the asphalt were tested by CCTs (Fire Testing Technology, London, UK). The test sample size was 10 cm × 10 cm × 0.5 cm, the heat radiation intensity was set to 50 kw/m^2^, and the combustion selects the air atmosphere. Asphalt characterization used real heat release rate and smoke production rate under fire conditions.

### 2.4. Single Factor Test

Based on the laboratory’s preliminary test foundation and reference to related research results [8,9,45], the value ranges of ADP and ATH are limited to 3~5 wt%. Within this range, the flame retardant can absorb the light components in the asphalt to increase the stability of the asphalt and improve the high temperature performance. When the addition amount of the flame retardant exceeds a certain range, the powder cannot be completely dispersed in the asphalt, and it may agglomerate. The result of which is a decrease in performance degradation. According to the literature [25], the dosage of halloysite nanotubes is limited to 2 wt%. Adding excessive amounts of HNTs will cause them to agglomerate, fail to exert their effects, and degrade road performance.

### 2.5. RSM Test Optimization Design Plan

This experiment relies on a single factor test and compounded HNTs and CFR modified asphalt. This study mainly considers the influence of the ATH content (wt%), ADP content (wt%), and HNTs content (wt%) of the flame retardant on the physical properties (penetration $Y_{pen}$, softening point $Y_{SP}$ and ductility $Y_{D}$) and flame retardant performance of the asphalt (oxygen index $Y_{LOI}$ and ignition point $Y_{IP}$). Using the Box-Behnken (BBD) model in Design Expert 11 software, a three-factor and three-level experimental design was carried out on the composition parameters of the flame retardant and a mathematical regression model was established. Find the most compounded amount of flame retardant through the model. The experimental factors and level range data are shown in Table 2. Choose 3 center points, a total of 17 sets of tests, the test plan and results are shown in Table 3.

## 3. Results

Through regression fitting analysis on the experimental data of physical properties, and the resulting regression models are as follows: Equations (1)–(3)

(1) $$Y_{pen}=264.64-82.74A-64.29B-37.13C+18.48AB\;+18.55AC-0.28BC+7.68A^{2}+6.33B^{2}-A^{2}B-2.23A^{2}C-1.38AB^{2}$$

(2) $$Y_{SP}=93.21-4.60A-4.42B+17.16C\;+0.88AB-2.35AC-0.15BC+0.51A^{2}+0.16B^{2}-1.57C^{2}$$

(3) $$Y_{D}=81.15-11.08A-15.20B-5.18C\;+1.75AC+0.50BC+1.03A^{2}+1.78B^{2}+0.53C^{2}$$

In the equations: 3 wt% ≤ *A* ≤ 5 wt; 3 wt% ≤ *B* ≤ 5 wt; 1 wt% ≤ *C* ≤ 2 wt%

Through regression fitting analysis on the experimental data of flame retardant performance, and the regression models obtained are as follows: Equations (4) and (5)

(4) $$Y_{LOI}=28.37-2.41A+1.02B+0.45C\;+0.05AB-0.10AC+0.38BC+0.31A^{2}+0.18B^{2}-0.47C^{2}$$

(5) $$Y_{IP}=663.68-82.40A-46.90B+9.65C\;+1.75AB+1.25AC+0.75BC+10.18A^{2}+5.18B^{2}-3.33C^{2}$$

In the equations: 3 wt% ≤ *A* ≤ 5 wt; 3 wt% ≤ *B* ≤ 5 wt; 1 wt% ≤ *C* ≤ 2 wt%

### 3.1. Model Checking and Analysis

#### 3.1.1. Model Checking

The most important step in response surface analysis is to perform variance analysis on the fitted regression model and test the significance of the regression coefficients. Table 4 shows the analysis of variance (ANOVA) of the test results of $Y_{pen}$, $Y_{SP}$, and $Y_{D}$. The significance test is determined by the *F* test [34,46], the equation is as follows.

(6) $$F=\frac{S_{R}^{2}/f_{R}}{S_{E}^{2}/f_{E}}$$

Among them, $S_{R}^{2}$, $S_{E}^{2}$, $f_{R}$ and $f_{E}$ are regression sum of squares, residual sum of squares, regression degrees of freedom and residual degrees of freedom, respectively.

In the significance test of the regression coefficients of the model, the larger the *F* value, the smaller the *p* value (Prob(*p*) > *F* probability). The lower the probability that the hypothesis $H_{0}$ holds, the more important the regression coefficient, the more significant the model. It can be seen from Table 4 that the *F* values of the three regression models of $Y_{pen}$, $Y_{SP}$, and $Y_{D}$ are about 18.97, 117.61, and 12.20, and the corresponding *p* value is much smaller than the *F* value. The model is considered to have good significance and statistical significance; The corresponding *p* values of the Lack of Fit are all greater than 0.05, indicating that the proportion of abnormal errors in the fitting of the obtained model is small. The fitting accuracy of the model is high, which can well describe the relationship between the response value and various factors.

Table 5 is the fitting statistical analysis table of the physical model. *R^2^* is the correlation coefficient of the model, and C.V. is the coefficient of variation. It indicates that the predicted value of the model obtains the scattering situation of the test point. The coefficient of variation is greater than 10%, indicating that there is a great change on the average level, and the model is unreasonable. The reliability analysis of the three models for physical performance optimization shows that the correlation coefficients of the three models of penetration, softening point, and ductility are 0.97, 0.99, and 0.94, respectively, which are all close to 1, and the coefficient of variation of the model is less than 10%, and the signal-to-noise ratio is greater than 4, which further illustrates the reliability of the model.

It can be seen from Table 6 and Table 7 that the *F* value of the limiting oxygen index is 17.05, the *p* value < 0.0001 is much smaller than the *F* value, and the regression correlation coefficient *R^2^* of the model is 0.93; the *F* value of the spontaneous ignition point is 9.18, the *p* value of 0.0040 is less than the *F* value, and the regression correlation coefficient *R^2^* of the model is 0.92. The significance of the two models is high, so these models can be used to make corresponding predictions.

#### 3.1.2. Model Analysis

**Figure 1:**
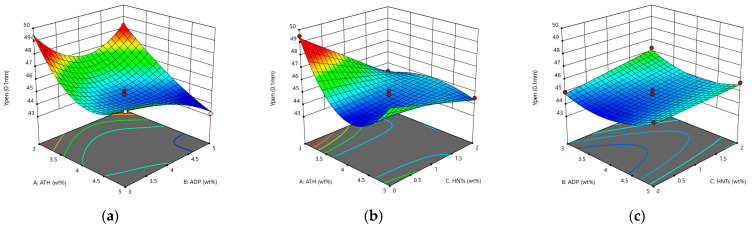
The response surface diagram of the flame retardant system to the change of penetration model: (**a**) Interactive effects of ATH and ADP; (**b**) Interactive effects of ATH and HNTs; and (**c**) Interactive effects of ADP and HNTs.

It can be seen from Figure 1 that the penetration model is a third-order model with an upward opening. Under the interaction of ATH, ADP, and HNTs, the penetration of asphalt shows a downward trend. Compared with ADP, ATH and HNTs have a more significant impact on the deterioration of penetration. This is mainly because ATH and HNTs are inorganic flame retardants, added to the asphalt to absorb light components to make the asphalt hard. ADP is an organic flame retardant, which can be well compatible with asphalt, and may strengthen the spatial network structure of the SBS modifier in asphalt.

**Figure 2:**
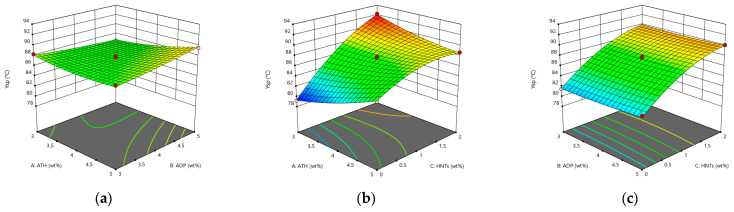
The response surface diagram of the flame retardant system to the change of softening point model: (**a**) Interactive effects of ATH and ADP; (**b**) Interactive effects of ATH and HNTs; and (**c**) Interactive effects of ADP and HNTs.

From Figure 2a, it can be seen that under the interactive influence of ATH and ADP, the softening point of asphalt first drops and then rises. When the content of ATH and ADP are both 4 wt%, it reaches the minimum value of 86.5 °C. The softening point reaches the maximum value of 89.5 °C when both are 5 wt%, and the difference between the minimum value and the maximum value is only 3 °C. It shows that the interactive influence of ATH and ADP does not have a great influence on the softening point. From Figure 2b,c, it can be seen that the softening point is increased obviously by HNTs. When HNTs changes from 0–1 wt%, the curvature of the curved surface changes more than when the content of HNTs changes from 1 wt% to 2 wt%, and when the content of HNTs is 0, it is the minimum softening point under the interactive effect. Therefore, the content of HNTs should be limited between 1 wt% and 2 wt%. After adding three kinds of flame-retardant materials, the softening point of asphalt was increased significantly, indicating that the three kinds of flame-retardant materials can improve the high temperature stability of asphalt. The reason for the increased high temperature stability is also related to the absorption of light components by the flame retardant.

**Figure 3:**
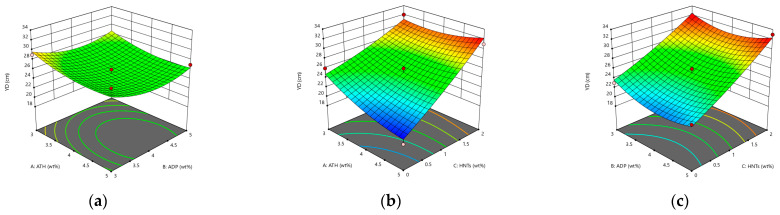
The response surface diagram of the flame retardant system to the change of ductility model: (**a**) Interactive effects of ATH and ADP; (**b**) Interactive effects of ATH and HNTs; and (**c**) Interactive effects of ADP and HNTs.

From Figure 3a,b, it can be seen that under the influence of the interaction, ATH reduces the ductility of the asphalt, and the single blending of ATH seriously deteriorates the low temperature performance of the asphalt. With the increase in the amount of ADP, the low temperature ductility of the asphalt decreases first and then increases. At the same time, HNTs can significantly increase the ductility of the asphalt. Among them, in the interaction with ATH, the increase of the ductility reaches 77.8% when the HNTs content changes from 0 to 2 wt%. It may be due to the well-dispersed flame retardant and SBS modifier that form a good spatial network cross-linked structure in the asphalt, the tubular structure of HNTs forms a reinforcement in the asphalt. In summary, there is no obvious deterioration of the low temperature ductility of the asphalt mortar modified by ATH, ADP, and HNTs.

**Figure 4:**
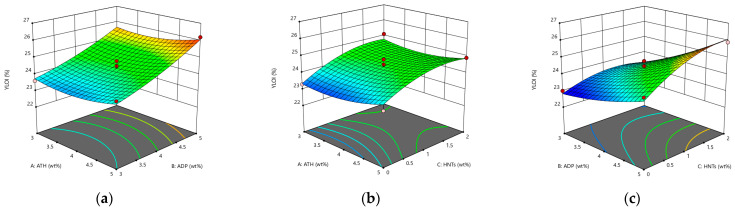
The response surface diagram of the flame retardant system to the change of LOI model: (**a**) Interactive effects of ATH and ADP; (**b**) Interactive effects of ATH and HNTs; and (**c**) Interactive effects of ADP and HNTs.

It can be seen from Figure 4 that the three flame retardant materials can increase the LOI of asphalt. Among them, HNTs does not improve significantly the flame retardant performance. The improvement of the flame retardant performance of the LOI composite modified asphalt is not obvious. The increase in the LOI is mainly due to the endothermic and flame-retardant effect of ATH, which competes with asphalt to absorb heat, making the asphalt insufficient to maintain continuous combustion at a low level, thereby increasing the LOI of asphalt.

**Figure 5:**
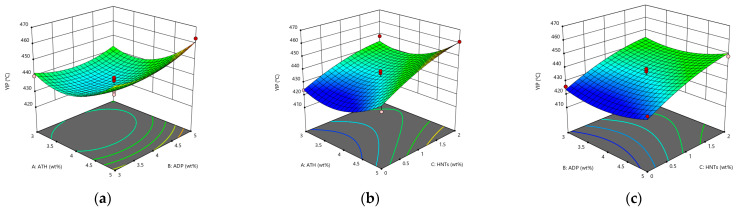
The response surface diagram of the flame retardant system to the change of ignition point: (**a**) Interactive effects of ATH and ADP; (**b**) Interactive effects of ATH and HNTs; and (**c**) Interactive effects of ADP and HNTs.

It can be seen from Figure 5 that ATH, ADP, and HNTs can increase IP of asphalt. The increase of IP of asphalt by ADP is not obvious compared with that of ATH and HNTs. This may be related to the flame-retardant mechanism of ADP. ATH can produce an endothermic flame retardant effect during the initial decomposition of asphalt, while the gas phase flame retardant effect of ADP mainly occurs in the second stage of asphalt combustion, and IP does not reach the decomposition temperature of ADP, so it shows that the change of ADP content does not significantly increase IP of the asphalt.

### 3.2. Formula Composition Optimization Based on the Normalized Value of the Overall Performance Evaluation

Based on the fitting model of asphalt physical properties (Penetration, Softening point, Ductility) and flame retardant properties (LOI, IP), in order to obtain comprehensively changes in physical properties and flame retardant properties, combining the desirability function to perform multi-objective optimization on the comprehensive performance of asphalt. Define that the performance indicators are converted into normalized values between 0–1, and then the product of the power exponents of the five indicators is calculated as the total evaluation normalized value OD. In Equation (7), $w_{i}$ is the weight of the *i*-th response value. Among them, in the physical properties of flame retardant modification, the smaller the penetration increment, the larger the softening point increment, the smaller the ductility attenuation, the better, and the larger the flame-retardant performance limit oxygen index and the ignition point increment, the better. Use $d_{min}$ in the Hassan method to transform the penetration $d_{1}$ and ductility $d_{2}$ to get the normalized value, and the softening point $d_{3}$ uses $d_{max}$ for conversion; the same limit oxygen index $d_{4}$ and spontaneous ignition point $d_{5}$ use $d_{max}$ for normalized conversion. In the Equations (8) and (9), Y is the value of the index; i is the experiment number; $Y_{max}$ is the maximum value of the index; $Y_{min}$ is the minimum value in the index.

(7) $$OD=d_{1}^{w1}d_{2}^{w2}d_{3}^{w3}d_{4}^{w4}d_{5}^{w5}$$

(8) $$d_{min}=(Y_{max}-Y_{i})/\left(Y_{max}-Y_{min}\right)$$

(9) $$d_{max}=\left(Y_{i}-Y_{min}\right)/(Y_{max}-Y_{min})$$

The normalized value (OD) results calculated by the general desire function are shown in Table 4. According to the data in Table 4, the multiple regression fitting analysis is performed, and the regression model equation of the OD value against the three factors of ATH, ADP, and HNTs is as follows.

(10) $$OD=1.47-0.01A-0.78B+0.05C-0.03AB+0.15AC-0.02BC\;+0.01A^{2}+0.13B^{2}-0.22C^{2}$$

In the equation: 3 wt% ≤ *A* ≤ 5 wt; 3 wt% ≤ *B* ≤ 5 wt; 1 wt% ≤ *C* ≤ 2 wt%

**Figure 6:**
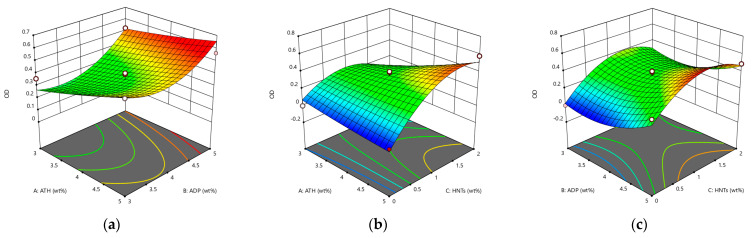
The response surface diagram of the flame retardant system to the change of OD value model: (**a**) Interactive effects of ATH and ADP; (**b**) Interactive effects of ATH and HNTs; and (**c**) Interactive effects of ADP and HNTs.

From the fitting variance analysis in Table 8, the *F* value of the model is 8.82. The model has good significance. From the individual significance test results, the order of significance of the influence of the three flame retardant materials on the normalized value of the general evaluation is: HNTs, ADP, and ATH. And the order of the influence of the interaction of each factor on the model is: AC > AB > BC, and the three-dimensional response surface of the interaction is shown in Figure 6.

The lack of fit of the model *p* = 0.1646 > 0.05, the lack of fit caused by the error is not significant, and the model’s determination coefficient *R^2^* = 0.9189, indicating that the correlation between the predicted value and the measured value is good.

It can be seen from Figure 6 that there is one and only one optimal solution for the OD value within the limited range, which is the maximum value. The optimal formula calculated by the model is ATH:ADP:HNTs = 3:5:1, and the total content is 9 wt%. The optimization of the flame retardant system formula has practical significance. According to the optimal solution given by the model, five parallel experiments were carried out to verify the accuracy of the optimization results. The average OD value obtained by the experiment was 0.430792, and the error with the predicted value was 0.002. It shows that the optimization of the model is reliable, and the optimal mixing ratio obtained is accurate.

### 3.3. Characterization of Composite Flame Retardant Modified Asphalt

The characterization of asphalt physical properties based on Penetration, Softening point, and Ductility and the characterization of flame retardant properties based on LOI and IP have certain limitations. In order to study the performance of asphalt in depth, MA, CFRMA (a flame retardant formulation of Test No.16), and HNTs-CFRMA were selected to analyze the high and low temperature performance of asphalt using rheological indicators. The emission characteristics and smoke emission characteristics of the asphalt were analyzed for the flame retardant performance.

#### 3.3.1. High Temperature Performance Characterization

1. Dynamic shear rheology test

**Figure 7:**
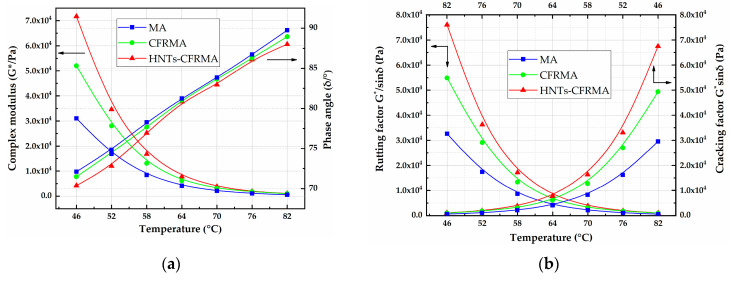
Characterization of flame retardant asphalt DSR-T high temperature performance: (**a**) Complex modulus $G^{*}$ and Phase angle δ; and (**b**) rutting factor $G^{*}/\sin\delta$ and cracking factor $G^{*}\sin\delta$.

In order to characterize the effect of flame retardants on the high temperature performance of MA, the rutting factor ($G^{*}/\sin\delta$) test and cracking factor ($G^{*}\sin\delta$) were carried out on MA, CFRMA, and HNTs-CFRMA with a dynamic shear rheometer. The complex shear modulus is the ratio of the maximum shear stress to the maximum shear strain, and the phase angle is the time lag between the applied force and the resulting strain. It can be seen from Figure 7 that at 46 °C, the complex modulus $G^{*}$ of MA, CFRMA, and HNTs-CFRMA are 31,042.1 Pa, 52,031.1 Pa and 71,638.5 Pa, respectively, and the phase angle δ is 72.11°, 71.49° and 70.38°, respectively. The factors ($G^{*}/\sin\delta$) are 32,619.3 Pa, 54,869.6 Pa and 76,054.2 Pa, and the cracking factors ($G^{*}\sin\delta$) are 29,541.2 Pa, 49,339.4 Pa and 67,479.2 Pa, respectively. Compared with MA and CFRMA, at the same temperature, the HNTs-CFRMA complex modulus increased by 40,596.4 Pa and 19,607.4 Pa, respectively. The phase angle decreased by 1.73° and 0.62°, and the rutting factor increased by 43,434.9 Pa and 21,184.6 Pa, and the cracking factor increased by 37,938.0 Pa and 18,139.8 Pa, respectively. It shows that adding flame retardant at the same temperature will make the asphalt change from soft to hard, the proportion of elasticity in the asphalt will increase, the viscosity component will decrease, and the high temperature performance will be better. The addition of HNTs may interact with the asphalt to form a stable network structure, which makes the asphalt better resistant to high temperature deformation, so that the complex modulus and rutting factor are higher than CFRMA.

With the increase of temperature, the complex modulus and rutting factor of MA, CFRMA, and HNTs-CFRMA decrease with the increase of temperature, and the phase angle increases with the increase of temperature. It shows that MA, CFRMA, and HNTs-CFRMA become softer with the increase of temperature, the difference of the asphalt’s complex modulus and rutting factor decreases, but the phase angle change law tends to be the same with the change of temperature.

2. Multiple stress creep test

**Figure 8:**
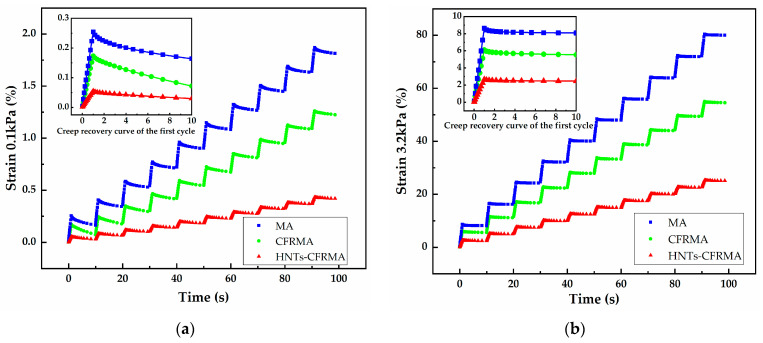
Characterization of flame retardant asphalt MSCR high temperature performance: (**a**) 0.1 kPa asphalt 10-cycle creep behavior curve; and (**b**) 3.2 kPa asphalt 10-cycle creep behavior curve.

In the multiple stress creep test, the load loading time is 1s, the unloading time is 9 s, and the repeated 10 cycles. During the test, the deformation of the asphalt during the loading stage will be partially recovered during the unloading stage, and the irreversible deformation will accumulate in the next loading cycle, so as to better simulate the process of repeated loading and unloading of different vehicle loads. Figure 8 shows the creep behavior of MA, CFRMA, and HNTs-CFRMA in 10 cycles under two loads.

In order to analyze the high temperature rheological properties of asphalt based on the MSCR, the deformation recovery rate R_@0.1_, R_@3.2_ and the irrecoverable creep compliance Jnr_@0.1_, Jnr_@3.2_ of the asphalt under the high and low stress levels were calculated. The calculation is shown in Equations (11) and (12).

(11) $$R=\frac{\gamma_{P}-\gamma_{nr}}{\gamma_{P}-\gamma_{0}}\times 100\%$$

(12) $$J_{nr}=\frac{\gamma_{nr}-\gamma_{0}}{\tau }\times 100\%$$

In the equation: $\gamma_{0}$ is the initial strain of each creep recovery period; $\gamma_{nr}$ is the residual strain after the recovery phase; $\gamma_{P}$ is the peak strain of each creep recovery period; τ is the loading stress, kPa; R is the deformation recovery rate; $J_{nr}$ is the irrecoverable creep compliance.

The calculation results of MA, CFRMA, and HNTs-CFRMA creep behavior parameters are shown in Table 9. The deformation recovery rates under two loads of 0.1 kPa and 3.2 kPa for 10 cycles are CFRMA, HNTs-CFRMA, MA in descending order. The deformation recovery rate of the same asphalt under 0.1 KPa is significantly higher than the deformation recovery rate of 3.2 KPa. The larger the deformation recovery rate, the stronger the elastic deformation ability of the asphalt material; on the contrary, the weaker the elastic deformation ability. This shows that the same kind of asphalt exhibits stronger elastic deformation ability under low stress, and the addition of flame retardant will make the elastic deformation ability of asphalt have a slight increase. Moreover, conventional flame retardants can improve the elasticity of asphalt more significantly than nanocomposite flame retardants. The non-recoverable creep compliance of MA, CFRMA, and HNTs-CFRMA under the load of 0.1 kPa and 3.2 kPa for 10 cycles is MA, CFRMA, HNTs-CFRMA in descending order. The irrecoverable creep compliance of the same asphalt at 0.1 kPa is significantly lower than the irrecoverable creep compliance of 3.2 kPa. Among them, the smaller the irrecoverable creep compliance, the smaller the unrecoverable deformation of the asphalt material, and the better the resistance to deformation under high temperature conditions; conversely, the worse the resistance to deformation under high temperature conditions. This shows that the same kind of asphalt exhibits strong resistance to deformation under low stress. After adding flame retardants, the asphalt’s ability to resist deformation is significantly improved under high temperature conditions, and the nanocomposite flame retardant enhances the asphalt’s high temperature resistance to deformation more significantly.

Based on the results of DSR-T and MSCR, the rutting factor and cracking factor of asphalt increase at the same temperature, while the irreversible creep compliance decreases, indicating that the high temperature rheological properties of asphalt are significantly improved. At the same time, HNTs-CFRMA has better high temperature rheological properties than CFRMA.

#### 3.3.2. Low Temperature Performance Characterization

1. Force ductility tester (FDT)

The force ductility test is used to characterize the low temperature performance of HNTs-CFRMA at 5 °C using tensile compliance f, yield strain energy E, and toughness ratio $R_{T/V}$. The calculation equation is as follows.

(13) $$f=D_{max}/F_{max}$$

(14) $$E=F_{max}\times D_{max}$$

(15) $$R_{T/V}=W_{T}/W_{V}$$

In the equation, $F_{max}$ represents the first stage (OA section), asphalt mainly exhibits the maximum tensile force when it is elastic; $D_{max}$ represents the displacement corresponding to the maximum tensile force; W is the viscous-toughness area, the area of the region enclosed by the stress-strain curve and the *X*-axis; $W_{V}$ is expressed as viscoelasticity, the second stage (AB section) of asphalt is shown as the area enclosed by the fitting straight line and the OA section and the abscissa in the decline stage of the yield characteristic, that is, the area of OAC; $W_{T}$ is the toughness, the area of the CBDE surrounded by the straight line and the curve on the right in the descending stage, $W_{T}=W-W_{V}$.

**Figure 9:**
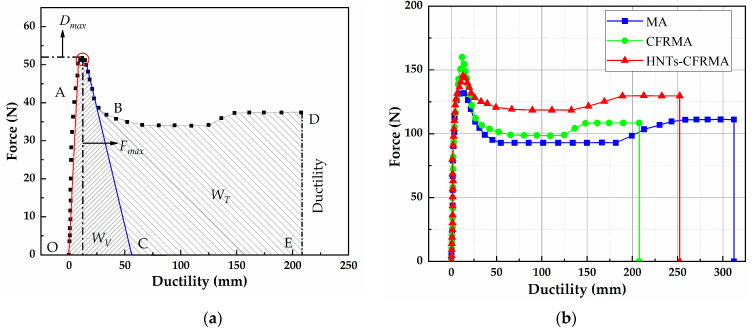
Characterization of asphalt low temperature performance based on force ductility: (**a**) Schematic diagram of force ductility; and (**b**) Force and displacement curve of asphalt.

It can be seen from Figure 9b that the low temperature ductility of MA is 312.28 mm, the low temperature ductility of CFRMA is 207.60mm, and the low temperature ductility of HNTs-CFRMA is 252.34 mm. Compared with MA, the ductility of HNTs-CFRMA is reduced by 59.94 mm, but compared with CFRMA, the ductility is increased by 44.74 mm. MA, CFRMA, and HNTs-CFRMA meet the ductility requirements of ASTM D 113, and the decrease in ductility may be caused by stress concentration caused by uneven dispersion of flame retardants in the asphalt.

It can be seen from Table 10 that the tensile compliance of HNTs-CFRMA is reduced by 0.0186 mm·N^−1^ compared with MA, and increased by 0.0120 mm·N^−1^ compared with CFRMA. The tensile compliance is the product of the maximum tensile force and the corresponding displacement, taking into account the dual factors of pull-down force and deformation at low temperature, which can well reflect the deformation ability of asphalt at low temperature. The larger the f, the stronger the deformability of the asphalt at low temperature and the better the low temperature performance. MA has the best low temperature performance, followed by HNTs-CFRMA, and CFRMA has the worst low temperature performance. Asphalt yield strain energy is the product of the maximum stress of the asphalt before yielding and the corresponding displacement. The larger the E, the greater the accumulated stress in the asphalt before the asphalt is stretched to yield, and the worse the asphalt’s stress relaxation ability. The yield strain energy of the three asphalts is CFRMA, HNTs-CFRMA, MA, in order, indicating that CFRMA has the worst stress relaxation ability, followed by HNTs-CFRMA, and MA has the best stress relaxation ability.

Combined with the toughness ratio of asphalt during the entire tensile fracture process, it is indicated that the addition of flame retardants makes the low temperature performance of asphalt weaker, and the effect of conventional flame retardants on asphalt deterioration is more obvious, and the addition of HNTs reduces the effect of deterioration.

2. Bending beam rheometer

Using the TE-BBR bending beam rheometer, the creep stiffness (S) and creep rate (m) of the three asphalt mortar beams at temperatures of −24 °C, −18 °C, and −12 °C were tested and calculated.

**Figure 10:**
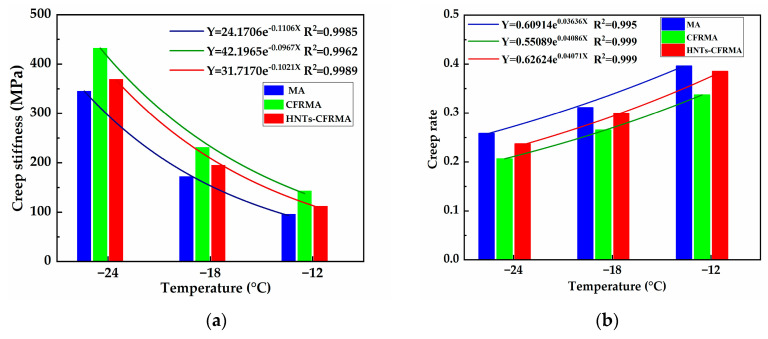
Variation law of asphalt creep characteristic parameters at low temperature: (**a**) Creep stiffness (S); and (**b**) Creep rate (m).

It can be seen from the Figure 10 and Table 11, S of MA, CFRMA, and HNTs-CFRMA gradually decreases with the increase of temperature (T). S and T can be fitted into a highly correlated exponential relationship. S of the asphalts at −24 °C, −18 °C, and −12 °C is MA, HNTs-CFRMA, and CFRMA, in descending order. S of asphalt indicates the degree of softness and hardness of the asphalt. The larger the S, the smaller the strain response value of the asphalt under a constant load. The asphalt exhibits brittleness and deteriorates low temperature performance. According to m of asphalt at low temperature, m of the asphalts gradually increases with the increase of T. m and T can be fitted into a linear relationship with higher correlation. m of the asphalts at the three temperatures are MA, HNTs-CFRMA, and CFRMA in descending order. With the increase of T, the difference between HNTs-CFRMA and MA decreases while the difference from CFRMA becomes larger. m of asphalt indicates the ability of asphalt to deform. The greater the m, the greater the deformation of the asphalt under load, the asphalt exhibits plasticity, and the low temperature flexibility is better. The ratio of m/S decreases with the increase of temperature. At the same temperature, the m/S of the asphalts are MA, HNTs-CFRMA, and CFRMA in descending order. The larger the ratio of m/S, the stronger the anti-cracking ability of the asphalt at low temperature. The result is consistent with the single index m and S to evaluate the performance of asphalt. This shows that the addition of conventional flame retardants will significantly degrade the low temperature performance of asphalt, while the addition of nanocomposite flame retardants can slightly improve the low temperature performance of asphalt, and the improvement effect decreases as the temperature decreases.

Comprehensive test results and analysis of FDT and BBR show that the low temperature rheological properties of asphalt are significantly degraded after adding conventional flame retardants, and the addition of nanocomposite flame retardants can make up for the deterioration of the low temperature rheological properties of asphalt by conventional flame retardants.

#### 3.3.3. Characterization of Flame Retardant Performance of Composite Modified Asphalt

**Figure 11:**
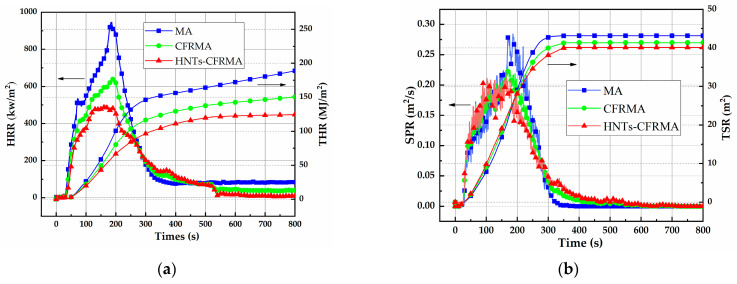
Characterization of flame retardant performance of flame retardant modified asphalt: (**a**) Heat release rate and total heat release; and (**b**) Smoke production rate and total smoke release.

In order to characterize the flame retardant properties of asphalt, CCTs was used to test the heat release rate and smoke production rate, heat release amount and smoke production. It can be seen from Figure 11a that MA is ignited at 21 s and the heat radiation intensity is 50 kw/m^2^, and the heat release rate rises sharply after ignition, reaching a peak value of 993.1 kw/m^2^. The ignition time of CFRMA and HNTs-CFRMA was 28 s and 30 s, respectively, which was extended by 33.3% and 42.9%, respectively. The heat release rate of CFRMA and HNTs-CFRMA increases slowly compared with MA, and the peak values are respectively 602.7 kw/m^2^ and 484.1 kw/m^2^, which are reduced by 390.4 kw/m^2^ and 509.0 kw/m^2^ compared to MA.

At the same time, it can be seen from Figure 11b that the peak smoke release rate is reduced from 0.279 m^2^/s of MA to 0.227 m^2^/s and 0.211 m^2^/s, respectively, compared with MA, which is reduced by 0.052 m^2^/s and 0.068 m^2^/s, respectively. At the 800th s, the total heat release and total smoke release of HNTs-CFRMA are 124.4 MJ/m^2^ and 40.14 m^2^, respectively. Compared with MA’s 188.6 MJ/m^2^ and 43.15 m^2^, it is reduced by 34% and 7%, and compared with CFRMA’s 150.1 MJ/m^2^ and 41.30 m^2^, it is reduced by 17% and 3%.

It shows that both CFRMA and HNTs-CFRMA can prolong the time that asphalt is ignited, and reduce the heat release rate, total heat release, smoke production rate, and total smoke release. The combination of HNTs and CFRMA can exert a synergistic enhancement effect to significantly reduce the thermal effect of asphalt combustion and reduce the generation of toxic smoke.

## 4. Conclusions

(1) The physical performance analysis of asphalt modified by nanocomposite flame-retardant shows that the HNTs reduces the ductility deterioration, reduces penetration, and has a greater impact on the physical properties of asphalt. The flame retardant performance analysis shows that ATH plays a major role in endothermic in the initial stage of thermal decomposition of asphalt, increasing the limiting oxygen index and ignition point and has a greater impact on the flame retardant properties of asphalt.
(2) A regression model was established based on response surface method multi-objective optimization. ADP has the most significant impact on the overall evaluation normalization value, because it mainly produces phosphoric acid to catalyze the condensation of asphalt into carbon and blocks the combustion process. The relative error is 0.21% measured by the response surface method between the predicted OD value and the actual value. Which indicates that the prediction effect of the model is accurate and effective, and the multi-objective optimization of comprehensive asphalt performance can be better achieved.
(3) Based on the rheological performance test and CCTs, MA, CFRMA, and HNTs-CFRMA were analyzed. The flame retardant compounded with HNTs and CFR is added to the modified asphalt to improve the high temperature performance of the asphalt. It compensates for the deterioration of the low temperature performance of asphalt caused by conventional flame retardants, and reduces the heat release rate and smoke release rate. It shows that it has good flame retardant performance and smoke suppression performance. And it is a kind of asphalt flame retardant system with excellent performance, environmental friendliness, and bright prospects.

## Figures and Tables

**Table 1:** Basic performance indicators of asphalt.

Type	Penetration/(0.1 mm)	Softening Point/°C	Ductility/cm	Ignition Point/°C
Modified asphalt	51.9	71.5	32	360
standard	40–60	≥60	≥20 (5 °C)	≥230
Test standard	ASTM D 5 [37]	ASTM D 36 [38]	ASTM D 113 [39]	AASHTO T 48 [42]

**Table 2:** Response surface three-factor three-level experimental design.

No.	Flame Retardant Material	Level One wt%	Level Two wt%	Level Three wt%
A	ATH	3	4	5
B	ADP	3	4	5
C	HNTs	0	1	2

**Table 3:** Box-Behnken optimized experimental design and results.

Test No.	SBS Modified Asphalt	ATH Dosage (wt%)	ADP Dosage (wt%)	HNTs Dosage (wt%)	Physical Properties			Flame Retardant Properties		OD
					Penetration 0.1 mm	Softening Point °C	Ductility cm	LOI %	IP °C	
SBS	100	0	0	0	51.9	71.5	35	18.1%	412	-
1	100	4	3	0	45.0	81.5	23	23.0%	426	0
2	100	5	5	1	43.2	89.5	27	26.2%	463	0.563451
3	100	5	3	1	46.3	87.8	29	24.1%	455	0.511977
4	100	3	4	2	44.3	92.2	32	25.1%	448	0
5	100	3	3	1	49.3	88.3	29	23.6%	440	0.358685
6	100	3	5	1	48.3	86.5	27	25.5%	441	0.523098
7	100	4	5	2	45.7	90.1	33	25.9%	448	0.490542
8	100	5	4	2	44.5	88.7	31	24.9%	461	0.581393
9	100	4	3	2	46.3	89.6	32	23.1%	442	0.241295
10	100	4	4	1	43.9	87.1	24	24.5%	438	0.397381
11	100	4	4	1	45.1	87.9	25	24.8%	437	0.394954
12	100	4	4	1	44.8	87.6	26	24.3%	439	0.404865
13	100	4	5	0	45.5	82.6	22	24.3%	429	0.316098
14	100	5	4	0	46.7	85.1	18	23.5%	432	0
15	100	4	4	1	44.2	87.3	26	24.3%	430	0.313444
16	100	3	4	0	49.5	79.2	26	23.3%	424	0
17	100	4	4	1	44.6	86.9	25	23.9%	428	0.25705

**Table 4:** Variance analysis of the physical performance fitting model.

Model	Source	Sum of Square	df	Mean Square	*F* Value	*p* Value	Statistical Significance
$Y_{pen}$	Model	52.66	11	4.79	18.97	0.0023	significant
	Lack of Fit	0.35	1	0.35	1.56	0.2798	not significant
$Y_{SP}$	Model	169.28	9	18.81	117.61	<0.0001	significant
	Lack of Fit	0.49	3	0.16	1.03	0.4693	not significant
$Y_{D}$	Model	236.01	9	26.22	12.20	0.0017	significant
	Lack of Fit	12.25	3	4.08	5.83	0.0607	not significant

**Table 5:** Response surface fitting statistical analysis of physical properties.

Model	*R^2^*	C.V. %	Adeq Precision
*Y_pen_*	0.97	1.12	14.85
*Y_sp_*	0.99	0.46	41.57
*Y_D_*	0.94	5.48	11.89

**Table 6:** Variance analysis of the flame retardant performance fitting model.

	Source	Sum of Square	df	Mean Square	*F* Value	*p* Value	Statistical Significance
$Y_{LOI}$	Model	0.00	3	0.00	17.05	<0.0001	significant
	Lack of Fit	0.00	9	0.00	2.53	0.1925	not significant
$Y_{SFP}$	Model	2049.24	9	227.69	9.18	0.0040	significant
	Lack of Fit	72.50	3	24.17	0.96	0.4947	not significant

**Table 7:** Response surface fitting statistical analysis of flame retardant performance.

Model	*R^2^*	C.V. %	Adeq Precision
*Y_LOI_*	0.93	1.33	14.62
*Y_SFP_*	0.92	1.13	9.75

**Table 8:** Analysis of the variance of OD value fitting model.

Source	Sum of Square	df	Mean Square	F Value	*p* Value	Statistical Significance
Model	0.62	9	0.07	8.82	0.0045	significant
A	0.08	1	0.08	9.61	0.0173	
B	0.08	1	0.08	9.77	0.0167	
C	0.12	1	0.12	15.91	0.0053	
AB	0.00	1	0.00	0.41	0.5432	
AC	0.08	1	0.08	10.82	0.0133	
BC	0.00	1	0.00	0.14	0.7165	
A^2^	0.00	1	0.00	0.05	0.8306	
B^2^	0.07	1	0.07	8.58	0.0220	
C^2^	0.20	1	0.20	25.56	0.0015	
Lack of Fit	0.04	3	0.01	2.91	0.1646	not significant
$R^{2}\;$= 0.9189						

**Table 9:** Characteristic parameters of asphalt creep behavior.

Type	R_@0.1_	R_@3.2_	Jnr_@0.1_	Jnr_@3.2_
MA	27.4101	5.9187	1.8	2.5
CFRMA	32.1751	8.8390	1.2	1.7
HNTs-CFRMA	30.2018	7.8829	0.4	0.7

**Table 10:** Asphalt-based low temperature performance index for force ductility.

**Type**	$F_{max}/N$	$D_{max}/\mathrm{mm}$	f/mm·N^−1^	E/N·mm	$R_{T/V}/\%$
MA	131.7	13.90	0.1055	1830.63	36.05
CFRMA	160.1	11.99	0.0749	1919.60	68.59
HNTs-CFRMA	145.5	12.65	0.0869	1840.55	61.48

**Table 11:** Characteristic parameters of asphalt creep at low temperature.

	Index	−24 °C			−18 °C			−12 °C		
Asphalt										
		S	m	m/S	S	m	m/S	S	m	m/S
MA		345	0.258	0.000748	172	0.311	0.001808	96	0.396	0.004125
CFRMA		432	0.206	0.000477	231	0.265	0.001147	143	0.337	0.002357
HNTs-CFRMA		369	0.237	0.000642	195	0.299	0.001533	112	0.385	0.003438
